# Detection of Inosine Monophosphate and the Umami Synergistic Effect Using a Taste Sensor with a Surface-Modified Membrane

**DOI:** 10.3390/molecules30214171

**Published:** 2025-10-23

**Authors:** Sota Otsuka, Mariko Koshi, Takeshi Onodera, Rui Yatabe, Toshiro Matsui, Kiyoshi Toko

**Affiliations:** 1 Graduate School of Information Science and Electrical Engineering, Kyushu University, 744 Motooka, Fukuoka 819-0395, Japan; otsuka.sota.551@s.kyushu-u.ac.jp; 2 Department of Bioscience and Biotechnology, Faculty of Agriculture, Graduate School of Kyushu University, 744 Motooka, Nishi-ku, Fukuoka 819-0395, Japan; 3 Faculty of Information Science and Electrical Engineering, Kyushu University, 744 Motooka, Fukuoka 819-0395, Japan; onodera@ed.kyushu-u.ac.jp (T.O.);; 4 Research and Development Center for Five-Sense Devices, Kyushu University, 744 Motooka, Fukuoka 819-0395, Japan; 5 Institute for Advanced Study, Kyushu University, 744 Motooka, Fukuoka 819-0395, Japan; 6 Food and Health Innovation Center, Nakamura Gakuen University, 5-7-1 Befu, Fukuoka 814-0198, Japan; 7 Graduate School of Nutritional Sciences, Nakamura Gakuen University, 5-7-1 Befu, Fukuoka 814-0198, Japan

**Keywords:** umami, taste sensor, lipid/polymer membrane, surface modification

## Abstract

A taste sensor composed of a lipid/polymer membrane using tetradodecylammonium bromide (TDAB) as the lipid and modified with 2,6-dihydroxyterephthalic acid (2,6-DHTPA) has recently been reported to exhibit high sensitivity and selectivity toward the umami substance monosodium L-glutamate (MSG). In this study, we aimed to investigate whether this sensor can also detect another umami substance, inosine monophosphate (IMP), and whether it can evaluate the umami synergistic effect—an enhancement of umami intensity—observed when IMP is mixed with MSG. Furthermore, ^1^H-NMR analysis was conducted to examine the nature of interactions between the membrane modifier and umami substances. The results demonstrated that IMP can be successfully detected using the sensor, and that, as previously reported for MSG, sensor sensitivity is influenced by the presence or absence of intramolecular hydrogen bonding within the modifier and intermolecular hydrogen bonding between the modifier and the umami substance. In addition, the response to mixed solutions of MSG and IMP was greater than the sum of individual responses, indicating that the umami synergistic effect can be evaluated using the taste sensor. NMR measurements also revealed that the presence of the membrane modifier enhances the interaction between IMP and MSG, supporting the observed synergistic effect.

## 1. Introduction

Taste is one of the fundamental human senses for perceiving external stimuli, enabling the evaluation of the nutritional value of foods and beverages and the avoidance of potentially harmful substances [1,2]. Five basic taste qualities—sweetness, sourness, saltiness, bitterness, and umami—are recognized by humans. Umami is widely accepted as a savory taste, often described as meat-like, and its primary component, monosodium glutamate (MSG), was discovered in kombu (kelp) broth by Kikunae Ikeda [3,4]. Umami substances not only enhance the flavor of foods and stimulate appetite but have also been reported to contribute to reducing the risks of hypertension and cardiovascular disease caused by excessive salt intake [5,6,7]. In addition to amino acid-based MSG, nucleotides such as inosine monophosphate (IMP) and guanosine monophosphate (GMP) are known umami substances. The combination of these substances is known to produce a pronounced enhancement of umami, a phenomenon referred to as the synergistic effect of umami [4,8,9,10,11,12,13]. A method to evaluate this synergistic effect, closely resembling biological taste response, has been reported using the mouse taste organoids with flow cytometry technique, which employs mouse taste bud organoids as sensing elements: its results have been shown to correlate well with sensory evaluations [14]. Commercially available electronic tongues or taste sensors cannot measure the synergistic effect of umami. In addition to sensory tests, various analytical techniques have been used to detect umami substances, including spectroscopic methods [15] as well as electronic tongues and taste sensors, which are superior from the viewpoint of the stability for methods utilizing organoids.

Taste sensing systems are known as electronic tongues or taste sensors. Various technologies have been employed for taste sensing, including potentiometric measurement [16,17,18,19,20,21,22,23,24,25,26,27], biosensing using biological tissues [28,29,30], and enzyme-based sensing methods [31,32,33]. Among these, taste sensors based on potentiometry using lipid/polymer membranes have been developed to evaluate various taste qualities by responding to the physicochemical properties of substances, such as charge and hydrophobicity [34,35]. These taste sensors have attracted attention as objective and highly reproducible tools for quality control and new product development in the food, beverage, and pharmaceutical industries [36,37,38,39,40,41,42,43,44,45,46,47,48]. For instance, when detecting bitter substances that are hydrophobic and carry a charge, taste sensors exhibit changes in membrane potential due to the adsorption of the bitter substances onto the membrane via electrostatic and hydrophobic interactions, leading to changes in the surface charge density. However, a fundamental limitation of potentiometric taste sensors is that they can generally detect only substances that are charged or ionizable.

However, it has been reported that a taste sensor can detect uncharged bitter substances, such as caffeine and theobromine, by modifying the surface of the lipid/polymer membrane [49]. In this method, 2,6-dihydroxybenzoic acid (2,6-DHBA) was used as a surface modifier. The transition from intramolecular to intermolecular hydrogen bonding within the modifier molecule increases the surface charge density of the membrane in the positive direction, leading to an increase in membrane potential. The hydroxyl group on the benzene ring can form intramolecular hydrogen bonding with the adjacent carboxyl group [50,51]. The interaction between 2,6-DHBA and caffeine was confirmed by nuclear magnetic resonance (NMR) analysis [52]. NMR spectroscopy provides detailed information on molecular structure and chemical environments by detecting the resonance frequencies of nuclei exposed to a magnetic field and pulsed radiofrequency irradiation [53]. The difference in resonance frequency, normalized relative to a standard, is referred to as the chemical shift (δ), which reflects the electron distribution in chemical bonds [54]. Therefore, by analyzing changes in the chemical shift, NMR spectroscopy can be used to investigate molecular-level interactions and bonding between substances. The development of taste sensors utilizing surface modification has demonstrated that even uncharged taste substances can be detected, thereby expanding the range of analytes measurable by taste sensors.

To evaluate umami taste, the conventional umami sensor AAE has been widely used [55,56,57,58]. In this sensor, the phosphoric acid di (2-ethylhexyl) ester (PAEE) lipid in the lipid/polymer membrane contains a phosphate group that donates a hydrogen ion to the carboxyl group of MSG, inducing a change in membrane potential and generating a negative response [59]. However, this type of sensor lacks strict selectivity for umami substances because a lipid PAEE also responds to astringent substances [60], indicating the need for improved discrimination capabilities based on the membrane’s molecular recognition of the chemical structure of MSG.

Although conventional taste sensors could not detect uncharged taste substances, a new surface modification method using 2,6-dihydroxyterephthalic acid (2,6-DHTPA) enabled the successful detection of caffeine, an uncharged bitter compound [49]. This membrane surface modification technique was subsequently applied to develop novel umami sensors capable of detecting MSG and sodium L-aspartate (MSA) [61,62,63]. These newly developed umami sensors demonstrated low responsiveness to the other basic tastes and exhibited high response selectivity toward MSG, enabling precise detection of umami substances.

Unlike conventional umami sensors employing the lipid PAEE, this umami sensor utilizes tetradodecylammonium bromide (TDAB) as the lipid component. TDAB is a fully dissociative lipid in aqueous solution and, unlike PAEE, is less susceptible to changes in pH. In measurements using TDAB-based umami sensors, it has been concluded that effective detection of umami substances requires the membrane modifier to possess an intramolecular hydrogen bond and two carboxyl groups, specifically arranged in a para-position. ^1^H-NMR analysis suggests that intermolecular interactions are formed between the modifier and the umami substance, leading to a change in the dissociation state of the modifier’s carboxyl groups. This causes an accumulation of protons on the membrane surface, thereby increasing the surface charge density in the positive direction, which ultimately produces a positive sensor response.

While these umami sensors have elucidated a detection mechanism based on molecular recognition, their focus has predominantly been on amino acid-based umami substances such as MSG. In contrast, this study aims to explore the sensing behavior of nucleotide-based umami compounds, specifically IMP. We investigated the structural requirements of membrane modifiers for effective IMP detection and examined the factors influencing sensor sensitivity. Additionally, we measured sensor responses to mixtures of MSG and IMP to determine whether the enhancement of umami via synergistic effects could be evaluated. To further elucidate the interaction mechanisms between membrane modifiers and umami substances, ^1^H-NMR spectroscopy was conducted.

## 2. Results and Discussion

### 2.1. Measurement of IMP Using a Taste Sensor with a 2,6-DHTPA-Modified Lipid/Polymer Membrane

**Figure 1:**
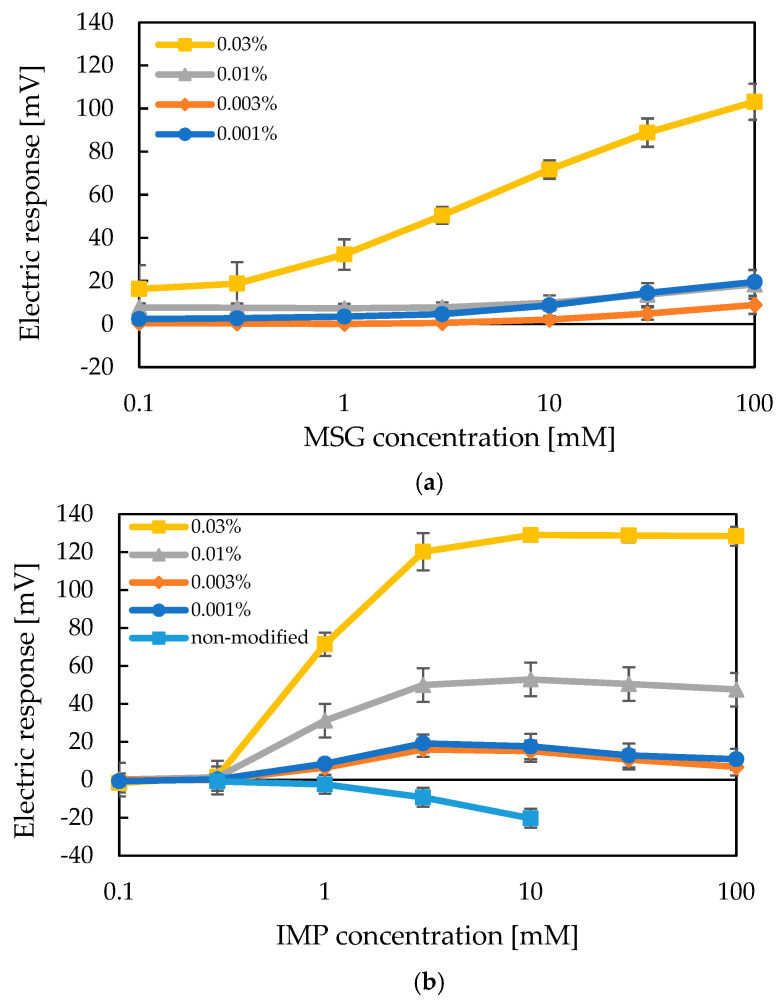
(**a**) Measurement results of MSG using taste sensors modified with varying concentrations of the modifier. (**b**) Measurement results of IMP using taste sensors modified with varying concentrations of the modifier.

First, we evaluated whether the umami sensor prepared with a lipid/polymer membrane modified with 2,6-DHTPA could detect IMP. 2,6-DHTPA contains two hydroxyl groups and one carboxyl group, and these adjacent functional groups can form intramolecular hydrogen bonds. In our previous study [61], it was shown that an umami sensor incorporating 1 mM of TDAB and modified with 0.03 wt% 2,6-DHTPA exhibited the highest response toward MSG. Furthermore, it was suggested that the molecular structure of the modifier significantly influences the sensor’s sensitivity to MSG. In the present study, we aimed to identify the optimal concentration of the modifier for detecting IMP and to discuss the structural requirements of the modifier necessary for IMP recognition, using the same sensor configuration as in the MSG detection. Lipid/polymer membranes containing 1 mM TDAB were modified with 2,6-DHTPA solutions at concentrations of 0.001, 0.003, 0.01, and 0.03 wt% for 72 h prior to measurement. The results of the sensor responses to both MSG and IMP are shown in Figure 1a,b. The detection limits of both MSG and IMP are low concentrations in the range of 0-point-something millimolar. These values are of the same order of the human sense [11,12,13].

As shown in Figure 1a, consistent with our previous report [61], the response to MSG increased with higher concentrations of the modifier. Figure 1b shows that the sensor response to IMP increased markedly with rising IMP concentration, but plateau at concentrations above 10 mM, suggesting saturation of the interaction between the modifier and IMP. Among the tested concentrations, the lipid/polymer membrane modified with 0.03 wt% 2,6-DHTPA exhibited the highest response, which agreed the results obtained for MSG. As found in Figure 1b, changing the concentration of the modifier only affected the saturation value of the response, while the midpoint remained unchanged around 1 mM IMP. It indicates that IMP and the modifier 2,6-DHTPA undergo a simple interaction.

**Figure 2:**
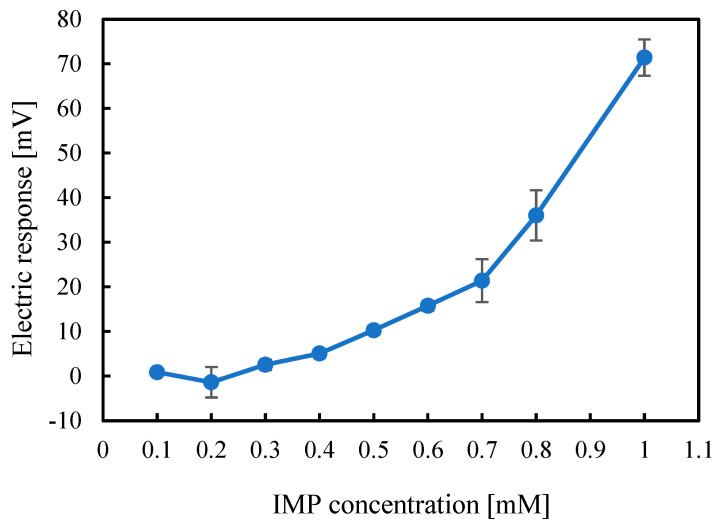
Sensor responses to low concentrations of IMP using a taste sensor with a 0.03 wt% 2,6-DHTPA-modified membrane.

Furthermore, Figure 1b indicates that the unmodified TDAB membrane exhibited a concentration-dependent response to IMP in the negative direction. Since a sharp increase in response was observed between 0.3 and 1 mM IMP, the results for this lower concentration range are presented in Figure 2.

The results of the measurements revealed that, in the low concentration range of IMP, the sensor response remained near 0 mV between 0.1 and 0.3 mM. Beyond this range, the response gradually increased with increasing IMP concentration. Taken together with the data shown in Figure 1b, it was confirmed that IMP elicited little to no response at concentrations between 0.1 and 0.3 mM but induced a sharp increase in response beyond that point. These findings indicate that modification of the lipid/polymer membrane with 2,6-DHTPA enables the umami sensor to exhibit high sensitivity toward IMP.

In previous studies using a lipid/polymer membrane modified with 2,6-DHTPA to detect MSG [61,62], the interaction between 2,6-DHTPA and MSG was explained as follows. The carboxyl group of 2,6-DHTPA forms an intramolecular hydrogen bond with the adjacent hydroxyl group, which facilitates the dissociation of a proton. The deprotonated carboxyl group of 2,6-DHTPA then forms an intermolecular hydrogen bond with the carboxyl group of MSG, thereby acting as a receptor for MSG. When this intermolecular hydrogen bond is formed between 2,6-DHTPA and MSG, the proton binds back to the carboxyl group of 2,6-DHTPA, resulting in a positive shift in the surface charge density of the membrane. Consequently, a positive sensor response is observed in the detection of MSG.

Based on the detection mechanism of MSG described above, the interaction between 2,6-DHTPA and IMP can be inferred as follows. The carboxyl group of 2,6-DHTPA dissociates a proton through the formation of an intramolecular hydrogen bond with its adjacent hydroxyl group. When IMP approaches, it forms an intermolecular hydrogen bond with 2,6-DHTPA. During this interaction, a proton binds back to the carboxyl group of 2,6-DHTPA, to result in a positive shift in surface charge density, thereby leading to a positive sensor response.

As with MSG detection, it is presumed that protons accumulate on the membrane surface due to the interaction between the membrane modifier and IMP, which is attributed to the specific structures of both molecules. This accumulation alters the surface charge density and allows the sensor to exhibit sensitivity toward IMP. To verify the existence of such intermolecular interactions between the modifier and IMP, ^1^H-NMR analysis was conducted in the following experiment.

### 2.2. Mechanistic Consideration of IMP Detection Using ^1^H-NMR Analysis

**Figure 3:**
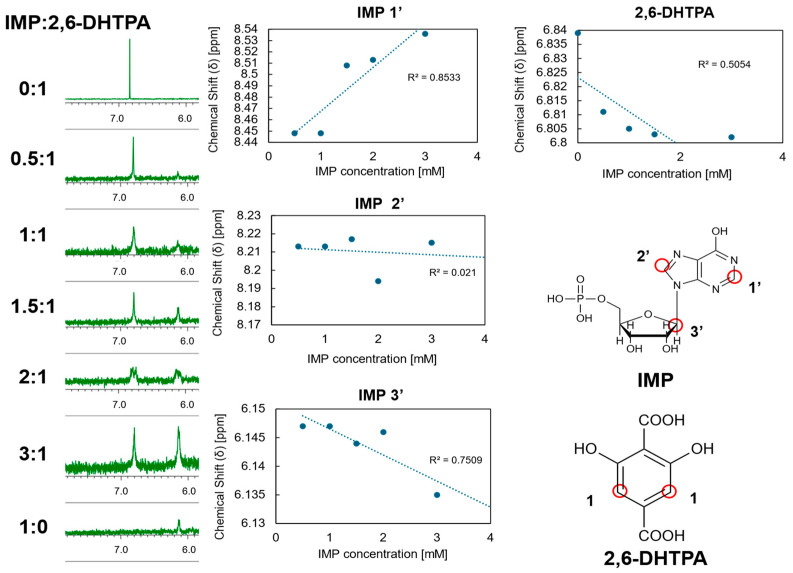
Spectra and chemical shift observed by ^1^H-NMR analysis with varying IMP concentrations. The red circles indicate the positions of hydrogens that showed chemical shift changes.

To analyze the nature of the intermolecular interactions between the membrane modifier and IMP, ^1^H-NMR spectroscopy was conducted. Figure 3 shows the chemical shift (δ) observed for the membrane modifier 2,6-DHTPA and the sample IMP as obtained from the ^1^H-NMR analysis.

As the molar ratio increased, the membrane modifier 2,6-DHTPA exhibited a notable change in its chemical shift, with the chemical shift value shifting from 6.839 to 6.805, corresponding of the change in chemical shift of 0.034. For IMP, significant chemical shift changes were also observed at the 1- and 3-positions of the hydrogen atoms, with changes in chemical shift values of 0.088 and 0.012, respectively. The presence of chemical shift changes in both the modifier and the sample, along with the positive sensor responses observed in the taste sensor measurements, suggest that 2,6-DHTPA forms bidirectional intermolecular interactions with IMP. The complete high-resolution NMR spectra covering the full chemical shift range are provided in the Supplementary Materials (Figure S1).

### 2.3. Measurement of Umami Synergistic Effect Using a Taste Sensor with 2,6-DHTPA-Modified Lipid/Polymer Membrane

**Figure 4:**
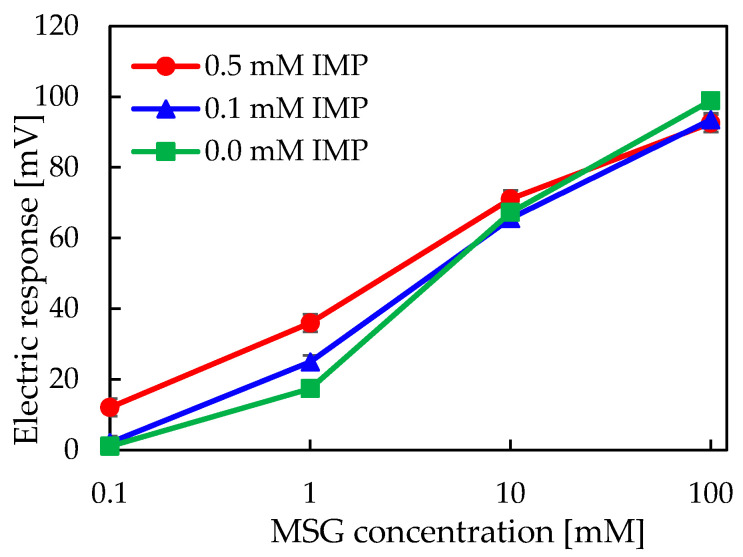
Measurement results for mixtures of MSG and IMP.

In this study, we investigated whether the enhancement of umami resulting from the umami synergistic effect [4,8,9,10] could be evaluated as an increase in sensor response. First, a taste sensor equipped with a lipid/polymer membrane modified with 2,6-DHTPA was used to measure MSG solutions prepared at concentrations of 0.1, 1, 10, and 100 mM. Next, solutions containing each concentration of MSG with the addition of IMP at 0.1 and 0.5 mM were measured. These results are shown in Figure 4, where “0.0 mM IMP” represents the measurement of MSG alone.

**Figure 5:**
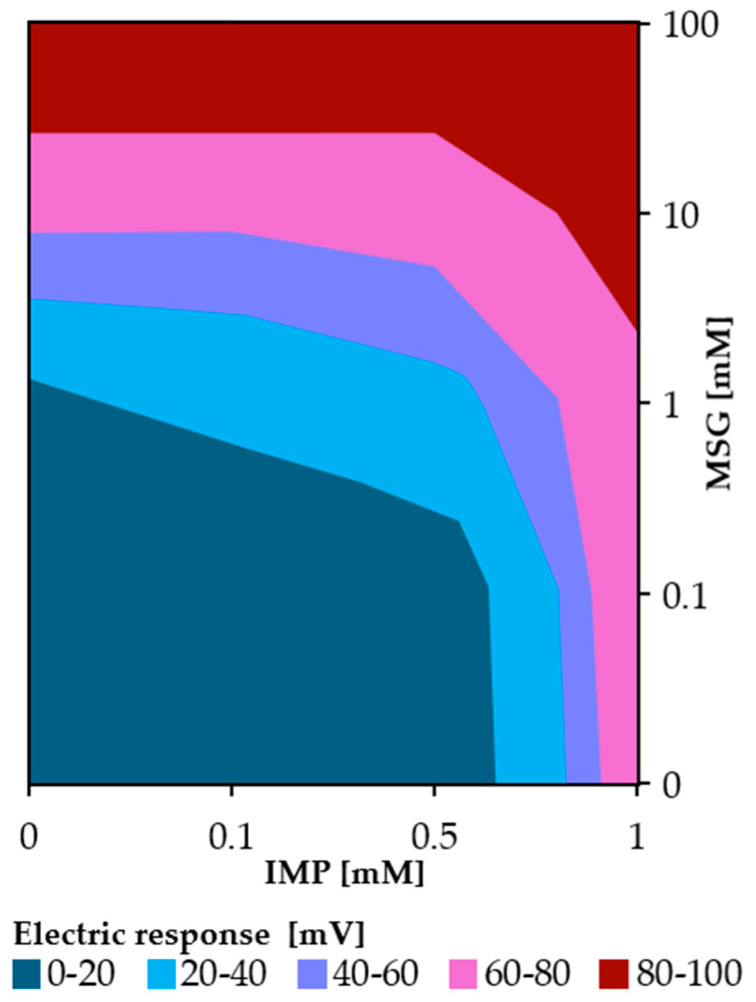
Contour map of taste sensor responses showing the umami synergistic effect between MSG and IMP.

Focusing on the data at 1 mM MSG, the sensor response for MSG alone was 17.3 mV, whereas the responses for the mixtures with 0.1 and 0.5 mM IMP were 24.9 mV and 35.9 mV, respectively. By subtracting the MSG-alone response from these values, the increases in response—defined as enhancement values—were calculated to be 7.6 mV and 18.6 mV, respectively. In contrast, the responses for 0.1 and 0.5 mM IMP alone were 0.9 mV and 10.3 mV, respectively. Since the enhancement values observed in the mixtures clearly exceeded the responses of the individual IMP solutions, these results indicate the presence of a synergistic effect between MSG and IMP. In Figure 5, a two-dimensional contour map is constructed by systematically varying the concentrations of MSG and IMP to evaluate the umami synergistic effect. We can see that the response increases with increasing MSG concentration at a certain IMP concentration such as 0.5 mM, while it increases with IMP concentration at certain value as 1 mM MSG. The resulting contour map clearly illustrates how the sensor response increases as a function of the combined concentrations, demonstrating the synergistic enhancement behavior over the measured range.

The positive response of the sensor was caused by protons returning to the membrane surface due to intermolecular hydrogen bonding between the membrane modifier and umami substances. In the mixed solution of MSG and IMP, the presence of dissociated IMP approaching the modified membrane creates a condition that facilitates the return of protons to the membrane surface. Consequently, an increased number of intermolecular hydrogen bonds are formed between MSG and the modifier 2,6-DHTPA, leading to an increased return to the membrane surface. It causes a more significant positive shift in the membrane surface charge density, thereby yielding a higher sensor response than that observed for MSG alone.

### 2.4. Consideration of the Mechanism for Measuring Umami Synergistic Effects Using ^1^H-NMR Analysis

**Figure 6:**
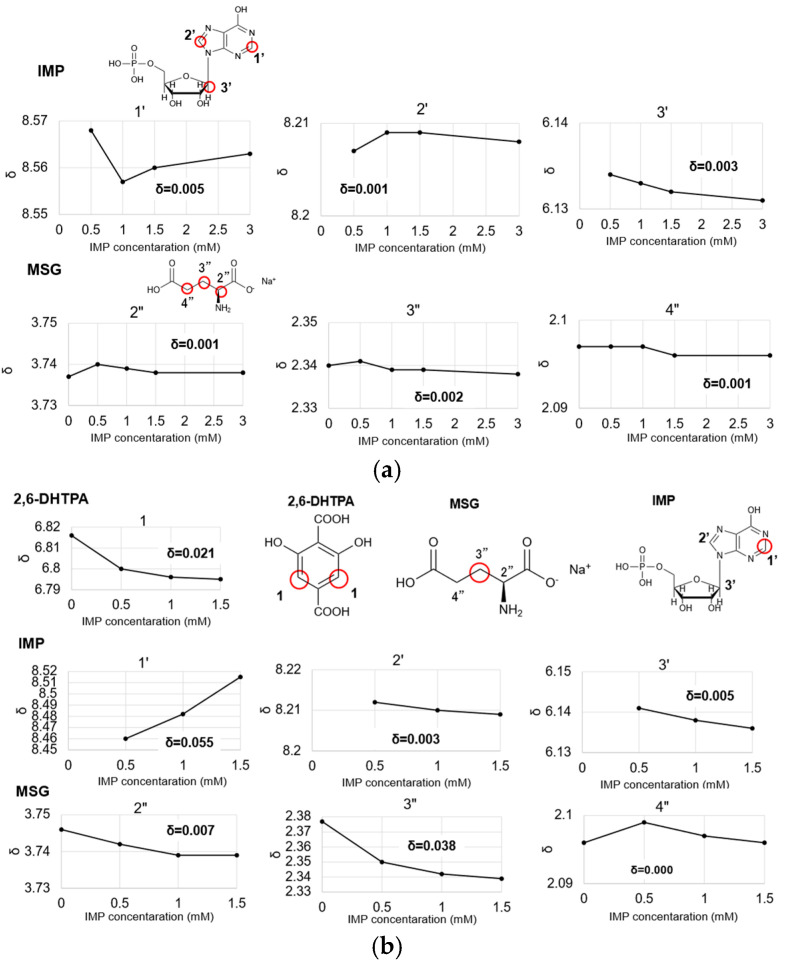
(**a**) Chemical shift changes of IMP and MSG analyzed by ^1^H-NMR. (**b**) Chemical shift changes of 2,6-DHTPA, IMP, and MSG analyzed by ^1^H-NMR. The red circles indicate the positions of hydrogens that showed chemical shift changes.

To analyze the nature of the intermolecular interactions occurring among the modifier, MSG, and IMP in a mixed solution, ^1^H-NMR analysis was performed. Figure 6 presents the chemical shift changes of each component—2,6-DHTPA (modifier), IMP, and MSG—when all three substances coexist, as well as the chemical shift changes observed when only IMP and MSG coexist in solution. Figure 6a,b shows that the chemical shift changes as the IMP concentration increases. The complete high-resolution NMR spectra covering the full chemical shift range are provided in the Supplementary Materials (Figures S2–S7).

As shown in Figure 6a, the maximum chemical shift change for IMP and MSG were approximately δ = 0.003, which is considered negligible and indicates the absence of significant chemical shift variations. In contrast, the presence of 2,6-DHTPA led to observable chemical shift changes in all three substances, with values one order of magnitude larger than those observed in the IMP and MSG binary mixture. This suggests that no interaction occurs between MSG and IMP alone, but the addition of 2,6-DHTPA enables interactions between MSG and IMP to become detectable. The fact that chemical shift changes were observed in all three components during coexistence is consistent with the positive response enhancement measured by the taste sensor. These results indicate that the membrane modifier, 2,6-DHTPA, facilitates indirect interactions between umami substances, thereby enabling the sensor to detect the synergistic umami effect.

### 2.5. A Proposed Model of Interaction Between IMP and 2,6-DHTPA

**Figure 7:**
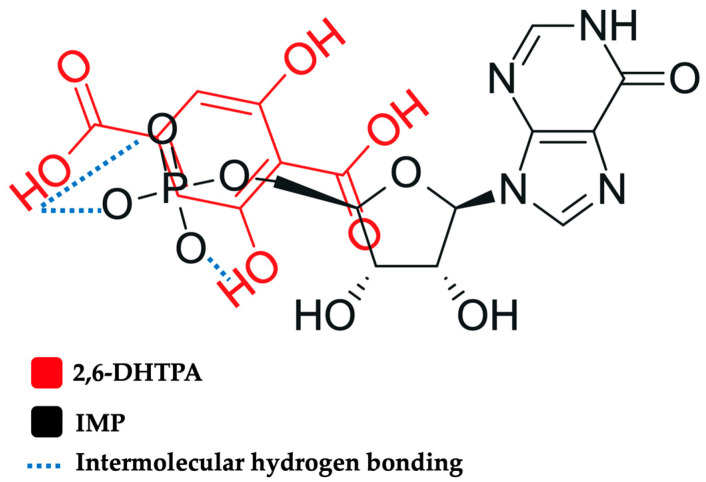
Proposed model of the interaction between IMP and 2,6-DHTPA. The red-colored and black molecules denote 2,6-DHTPA and IMP, respectively. Blue dashed lines imply intermolecular hydrogen bonding.

Based on the above NMR measurements, chemical-shift changes were observed for both IMP and 2,6-DHTPA, indicating that molecular interactions likely occurred between these compounds. In addition, the sensor exhibited a positive response to IMP. Therefore, it is reasonable to interpret that two protons returned to the carboxyl groups of 2,6-DHTPA, suggesting a transition from intramolecular hydrogen bonding within a 2,6-DHTPA molecule to intermolecular hydrogen bonding between IMP and 2,6-DHTPA. The dissociation constants, pKa1 and pKa2, of 2,6-DHTPA are pKa1 = 1.19 and pKa2 = 3.89, those of IMP being pKa1 = 1.53 and pKa2 = 6.25, calculated from Marvin 24.3.2 (ChemAxon, Budapest, Hungary). The change in electric charge becomes positive with the intramolecular to intermolecular hydrogen bonding. Figure 7 shows a molecular model of the hydrogen bonds between 2,6-DHTPA and IMP. This interpretation does not contradict previous findings in biological systems, where the phosphate group of IMP plays an essential role in molecular recognition in the gustatory receptor [64,65].

## 3. Materials and Methods

### 3.1. Reagent

TDAB (Sigma-Aldrich Japan G.K., Tokyo, Japan) was used as the lipid component of the lipid/polymer membrane. Polyvinyl chloride (PVC; FUJIFILM Wako Pure Chemical Corporation, Osaka, Japan) was employed as the polymer support, and dioctyl phenylphosphonate (DOPP; Dojindo Laboratories, Kumamoto, Japan) was used as the plasticizer. Tetrahydrofuran (THF; Sigma-Aldrich Japan G.K., Japan) was used as the organic solvent to dissolve these components.

2,6-DHTPA, obtained from BLDpharm (Shanghai, China), was used as the membrane modifier. Sodium hydroxide (NaOH) was purchased from FUJIFILM Wako Pure Chemical Corporation. MSG, IMP, sodium chloride (NaCl), potassium chloride (KCl), and tartaric acid were all purchased from Kanto Chemical Co., Inc., Tokyo, Japan. All reagents were used as received without further purification.

**Figure 8:**
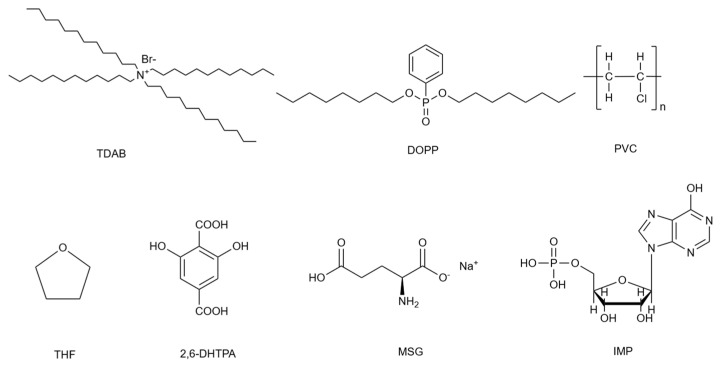
Chemical structures of TDAB, DOPP, PVC, THF, 2,6-DHTPA, MSG, and IMP.

The molecular structures of TDAB, DOPP, PVC, and THF used in the lipid/polymer membrane, as well as the modifier 2,6-DHTPA and the umami substances MSG and IMP used in the measurements, are shown in Figure 8.

### 3.2. Solutions

A reference solution was prepared by mixing 30 mM KCl and 0.3 mM tartaric acid, which was used to determine the baseline potential of the taste sensor. Sample solutions were prepared by dissolving MSG at concentrations of 0.1, 1, 10, 30, 100, and 300 mM, and IMP at concentrations of 0.1, 0.3, 1, 3, 10, 30, and 100 mM, into the reference solution. IMP was dissolved in the reference solution used for the measurements at concentrations ranging from 0.1 to 100 mM, and consequently the pH varied within the range of approximately 3.2 to 5.8.

For taste sensor measurements, the membrane was rinsed with a washing solution composed of 10 mM NaOH and 100 mM sodium chloride NaCl dissolved in distilled water.

### 3.3. Preparation of the Lipid/Polymer Membrane

In this study, sensor electrodes using a lipid/polymer membrane were employed for the taste sensor. The lipid/polymer membrane was prepared by dissolving 10 mM TDAB (1 mL), DOPP (1.5 mL), and PVC (800 mg) in 9 mL of THF. The resulting solution was poured into a 90 mm-diameter Petri dish, and the membrane was formed by evaporating the THF. The membrane was then dried in the lidded Petri dish at 25 °C (room temperature) for 24 h. After drying, the membrane was cut to approximately 10 mm × 6 mm and used for measurements. The thickness of the membrane used in this study was approximately 300 µm. The fabricated lipid/polymer membrane was cut into pieces and attached to the sensor probe using an adhesive solution prepared by dissolving 800 mg of PVC in 10 mL of THF.

### 3.4. Assembly of Electrodes and Surface Modification of the Lipid/Polymer Membrane

**Figure 9:**
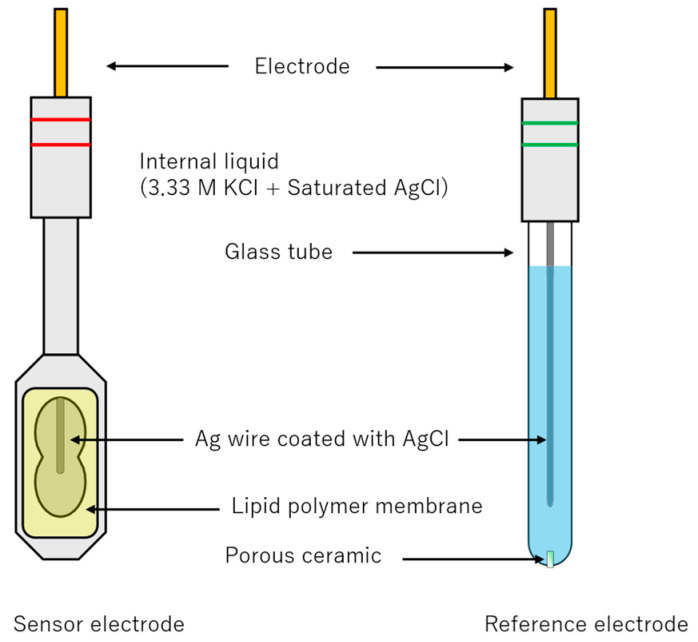
Structures of the sensor electrode and the reference electrode.

The sensor probe, to which the lipid/polymer membrane was attached, was filled with an internal solution containing 3.33 M KCl and saturated AgCl solution. A silver wire coated with AgCl was then inserted into the probe. The structures of the sensor and reference electrodes are shown in Figure 9.

Surface modification of the lipid/polymer membrane was performed using 2,6-DHTPA. Aqueous solutions of 2,6-DHTPA were prepared using ultrapure water as the solvent, at concentrations of 0.001, 0.003, 0.01, and 0.03 wt%. At concentrations above 0.03 wt%, 2,6-DHTPA could not be completely dissolved in water, and therefore higher concentration solutions could not be prepared. At such concentrations, 2,6-DHTPA was insoluble and could not be used to modify the lipid/polymer membrane. The assembled sensor electrodes were immersed in the prepared 2,6-DHTPA solutions for 72 h to modify the membrane surface. In addition, in Section 2.1, unmodified lipid/polymer membranes were also used for measurement.

### 3.5. Measurement of Umami Substances Using a Taste Sensor

Taste measurements in this study were performed using the taste sensing system TS-5000Z (Intelligent Sensor Technology, Inc., Kanagawa, Japan). The measurement procedure followed that described in our previous report [62]. The detection process for umami substances using the taste sensor consists of multiple steps. First, the sensor and reference electrodes shown in Figure 7 were immersed in the reference solution, and the reference potential *Vr* was recorded. Next, the electrodes were immersed in the sample solution containing umami substances, and the sample potential vs. was measured. The sensor response was defined as the potential difference between *Vr* and vs. Finally, the electrodes were immersed in a cleaning solution to restore the initial charge state of the membrane surface, allowing for the measurement of the next sample after obtaining both the reference and sample potentials. This cycle was repeated five times. To ensure data reliability, the average of the third to fifth measurements—where stable responses were observed—was used for analysis.

In our previous study on umami substance detection [63], it was shown in detecting MSG that the membrane modifier must possess a carboxyl group in the starting position and an additional carboxyl or hydroxyl group at the para- or meta-position on the benzene ring, enabling the formation of intramolecular hydrogen bonds. Upon immersion in the sample solution, the modifier on the membrane surface forms intermolecular hydrogen bonds with MSG. This transition from intra- to intermolecular hydrogen bonding causes protons to return to the carboxyl groups of the modifier, resulting in a shift in the membrane surface charge in the positive direction, thereby producing a positive sensor response. In contrast, unmodified membranes yield a negative response to MSG, indicating that surface modification enhances sensitivity and selectivity toward MSG detection.

This study investigated whether the MSG detection mechanism could be applied to IMP and used to evaluate their umami synergy.

### 3.6. ^1^H-NMR Measurement

In ^1^H-NMR measurements, the nuclear magnetic resonance of hydrogen nuclei in molecules is observed to determine molecular structure. The ^1^H-NMR spectra were recorded using an ECS-400 spectrometer (JEOL, Tokyo, Japan). The analyzed modifier was 2,6-DHTPA, and the sample was IMP. Mixed solutions of these two substances were prepared at molar ratios of IMP to 2,6-DHTPA of 0:1, 0.5:1, 1:1, 1.5:1, 2:1, and 3:1, D_2_O as the solvent. To match the ionic environment used in the taste sensor measurements, 1 mM KCl was added to all solutions. The ^1^H-NMR measurements were conducted under the following conditions: acquisition time of 2.73 s, 8 scans, a relaxation delay of 12 s, automatic gain adjustment, and a spinning rate of 15 Hz.

## 4. Conclusions

In this study, a taste sensor employing a lipid/polymer membrane modified with 2,6-DHTPA was used to detect the umami substance IMP and to evaluate the umami synergistic effect resulting from its mixture with MSG. The results demonstrated that IMP could be effectively detected using the 2,6-DHTPA-modified membrane, similarly to MSG. ^1^H-NMR analysis revealed that intermolecular interactions between IMP and the modifier are essential for detection.

Moreover, the sensor successfully evaluated the umami synergistic effect, as evidenced by a significantly greater response to MSG-IMP mixtures compared to the individual components. While no direct interaction between MSG and IMP was observed, ^1^H-NMR spectra indicated chemical shift changes in both substances only in the presence of 2,6-DHTPA, suggesting that the modifier facilitates indirect interactions between umami substances.

These findings confirm that the 2,6-DHTPA-modified taste sensor is not only effective for detecting individual umami substances, but also capable of evaluating synergistic effects between multiple umami compounds.

## Data Availability

The data presented in this research are available on request.

## Supplementary Materials

The following supporting information can be downloaded at: https://www.mdpi.com/article/10.3390/molecules30214171/s1, Figure S1: Full spectrum (IMP: 2,6-DHTPA); Figure S2: Full spectrum (2,6-DHTPA: MSG: added IMP); Figure S3: Expanded spectrum [δ 5–10 ppm] (2,6-DHTPA: MSG: added IMP); Figure S4: Expanded spectrum [δ < 5 ppm] (2,6-DHTPA: MSG: added IMP); Figure S5: Full spectrum (MSG: IMP); Figure S6: Expanded spectrum [δ 5–10 ppm] (MSG: IMP); Figure S7: Expanded spectrum [δ < 5 ppm] (MSG: IMP).
