# Supplementary material for: Detection of Inosine Monophosphate and the Umami Synergistic Effect Using a Taste Sensor with a Surface-Modified Membrane
Source: Molecules. 2025 Oct 23;30(21):4171. doi: 10.3390/molecules30214171 (PMC12609416; doi:10.3390/molecules30214171)
Supplement: Supplementary file 1 [file molecules-30-04171-s001.zip › molecules-3872976-supplementary.pdf]

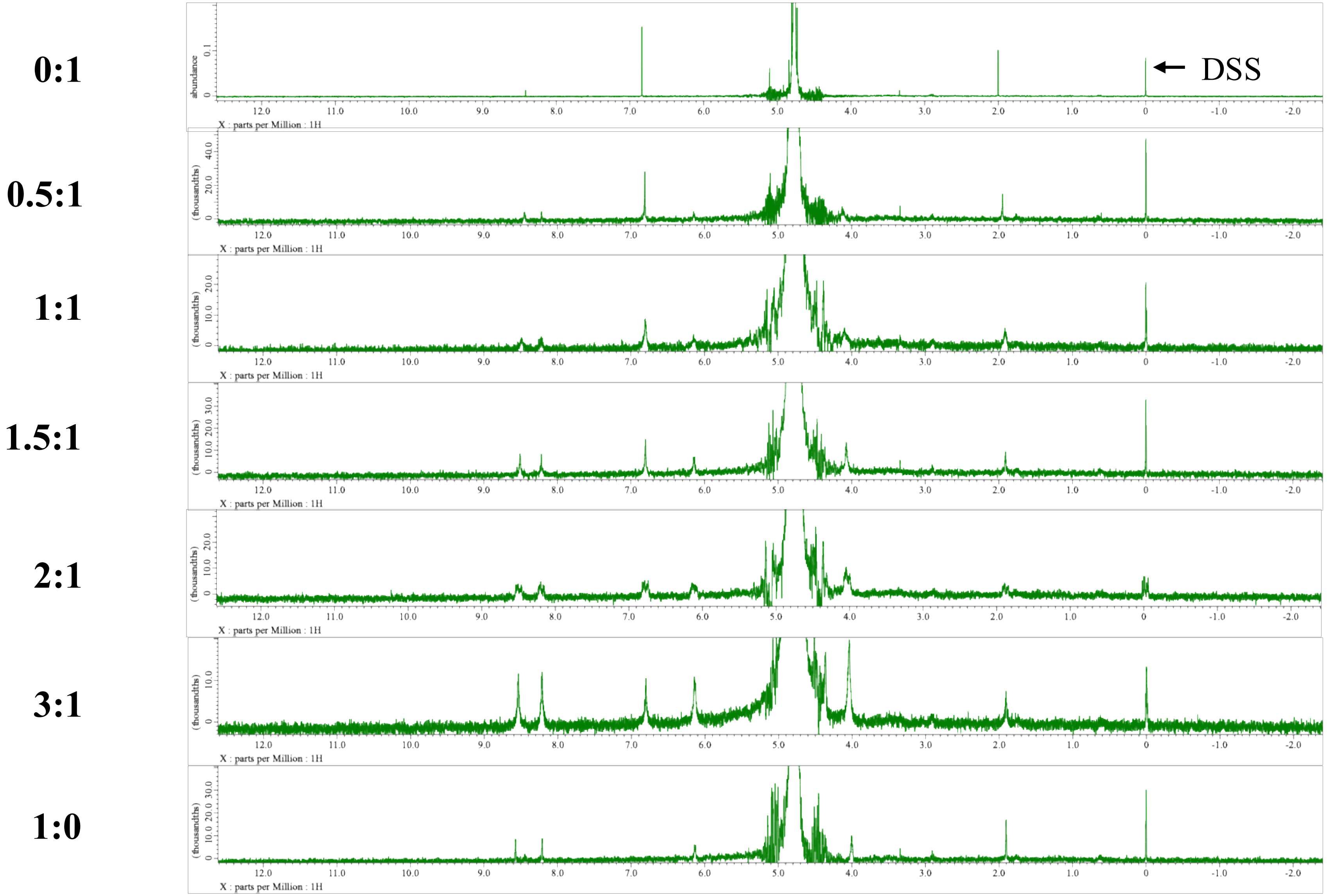

Figure S1. Full spectrum (IMP : 2,6-DHTPA)

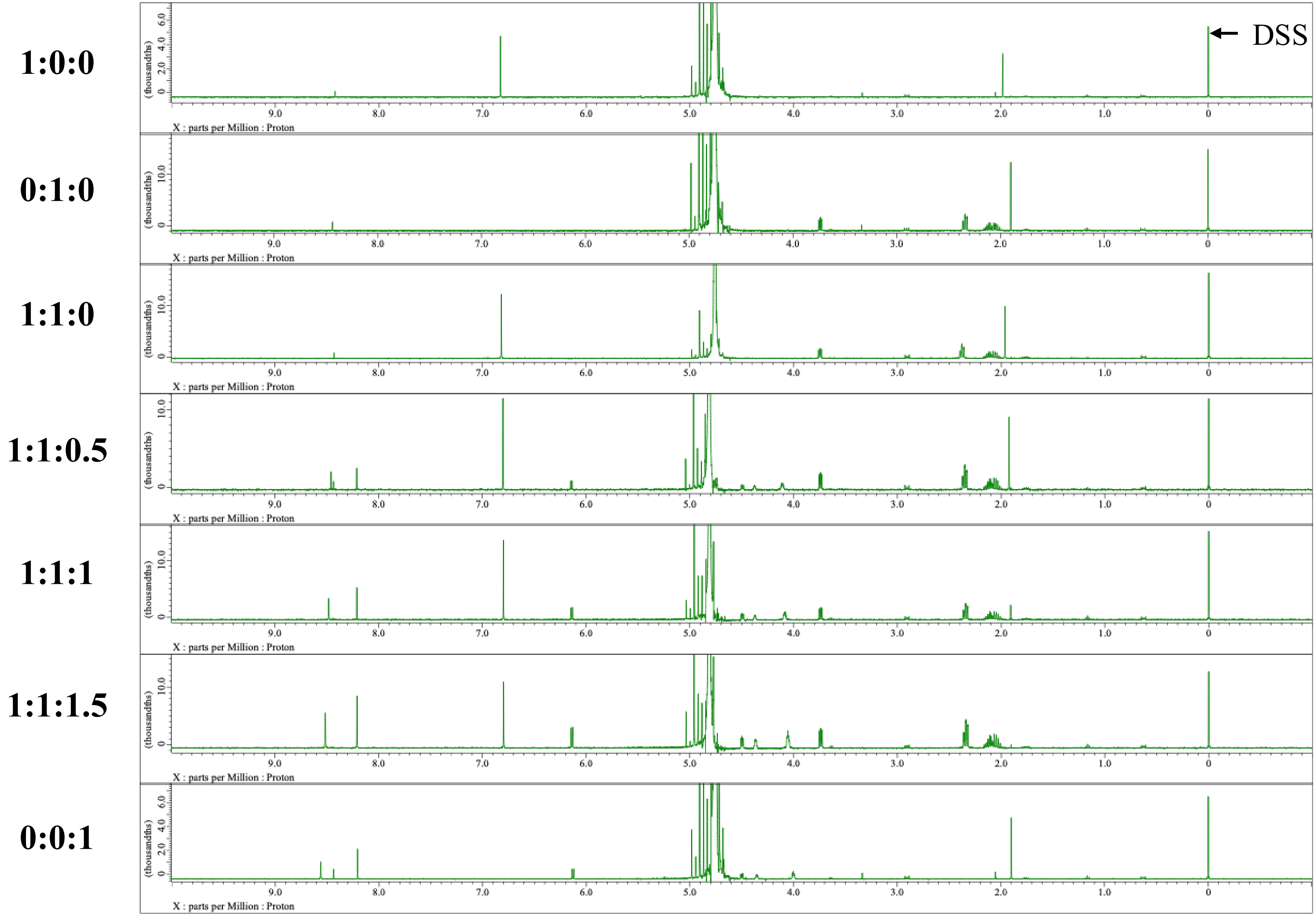

Figure S2. Full spectrum (2,6-DHTPA： MSG：added IMP)

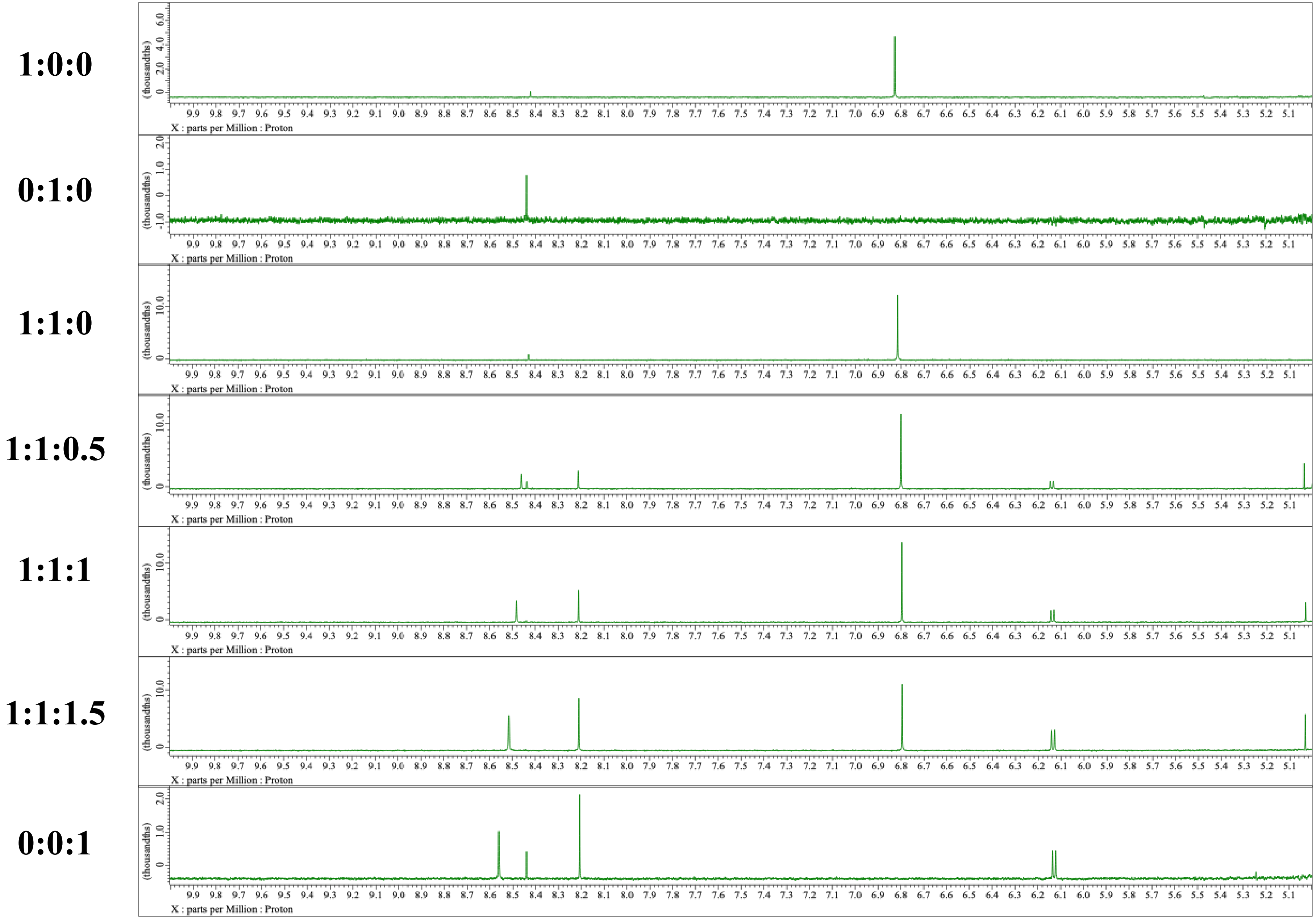

Figure S3. Expanded spectrum [δ 5–10 ppm] (2,6-DHTPA： MSG：added IMP)

1:0:0

0:1:0

1:1:0

1:1:0.5

1:1:1

1:1:1.5

0:0:1

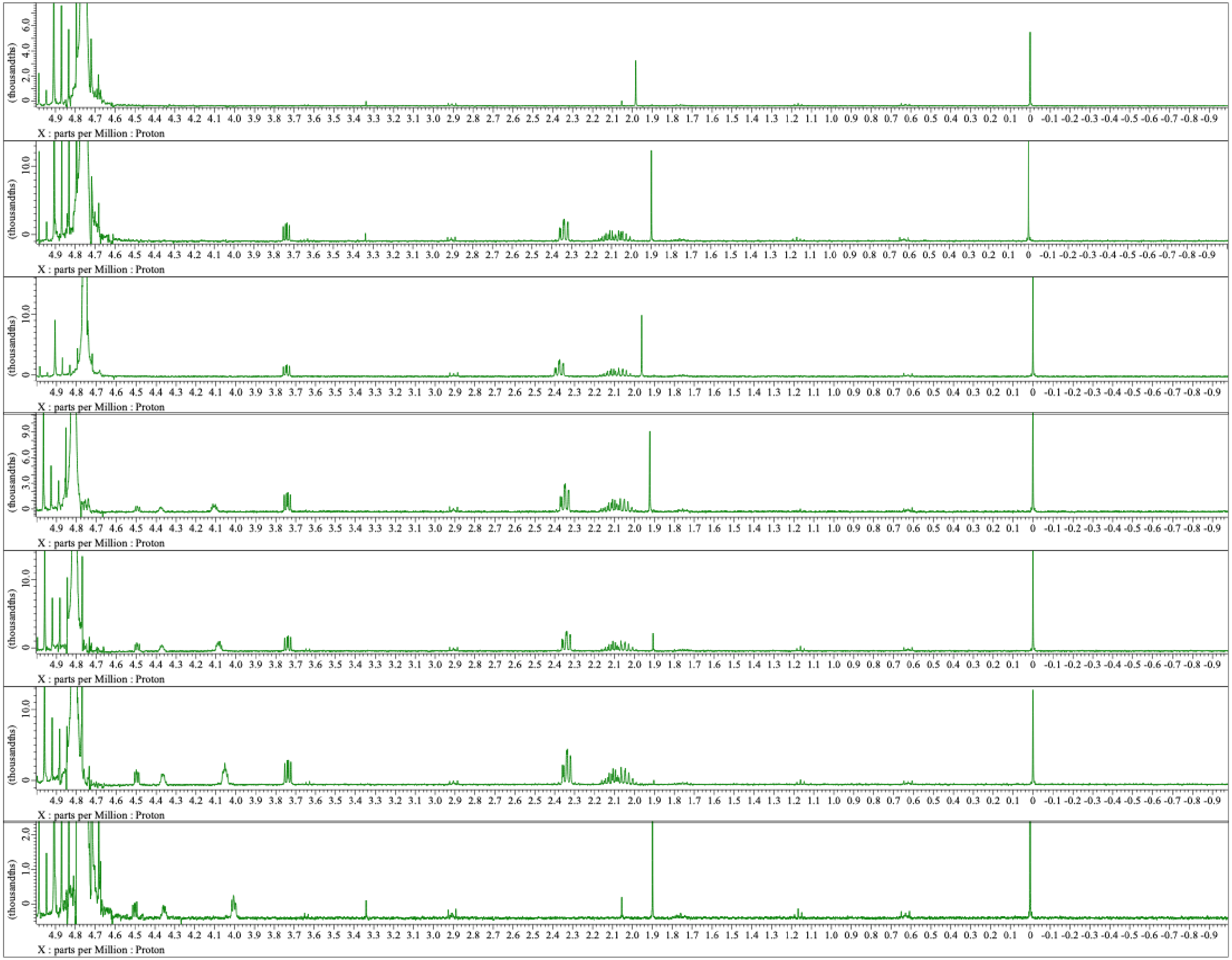

Figure S4. Expanded spectrum [δ < 5 ppm] (2,6-DHTPA : MSG : added IMP)

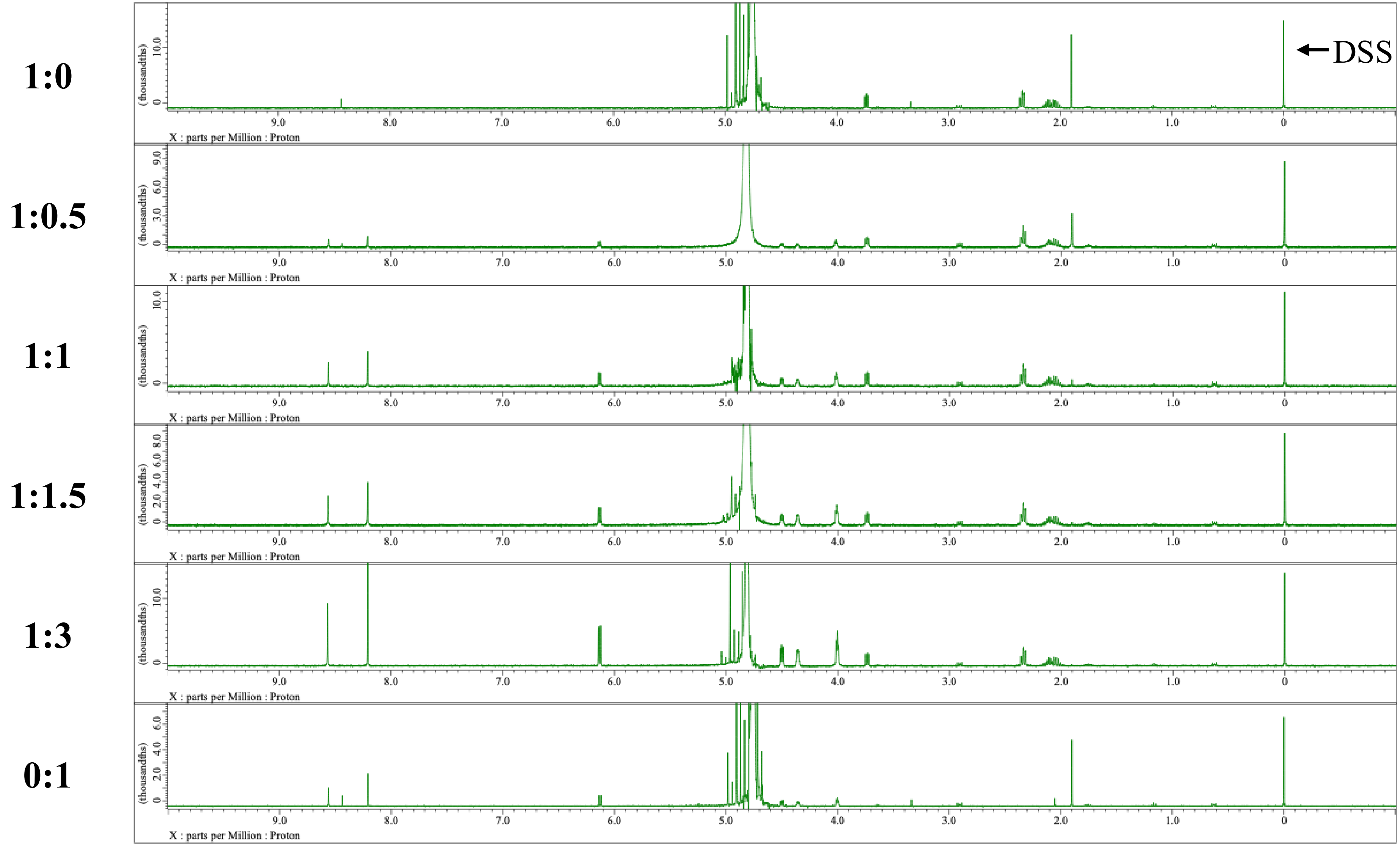

Figure S5. Full spectrum (MSG：IMP)

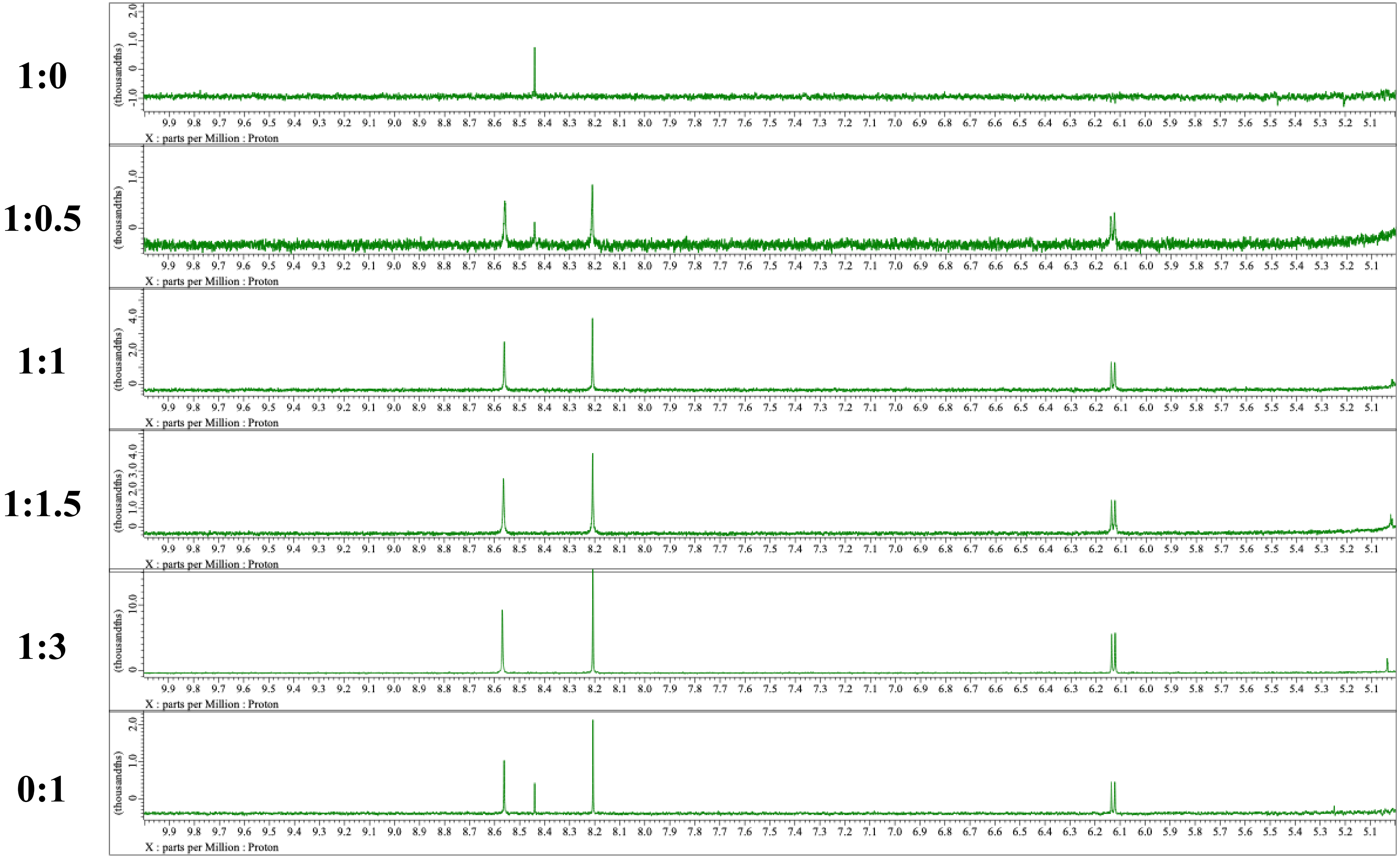

Figure S6. Expanded spectrum [δ 5–10 ppm] (MSG： IMP)

1:0

1:0.5

1:1

1:1.5

1:3

0:1

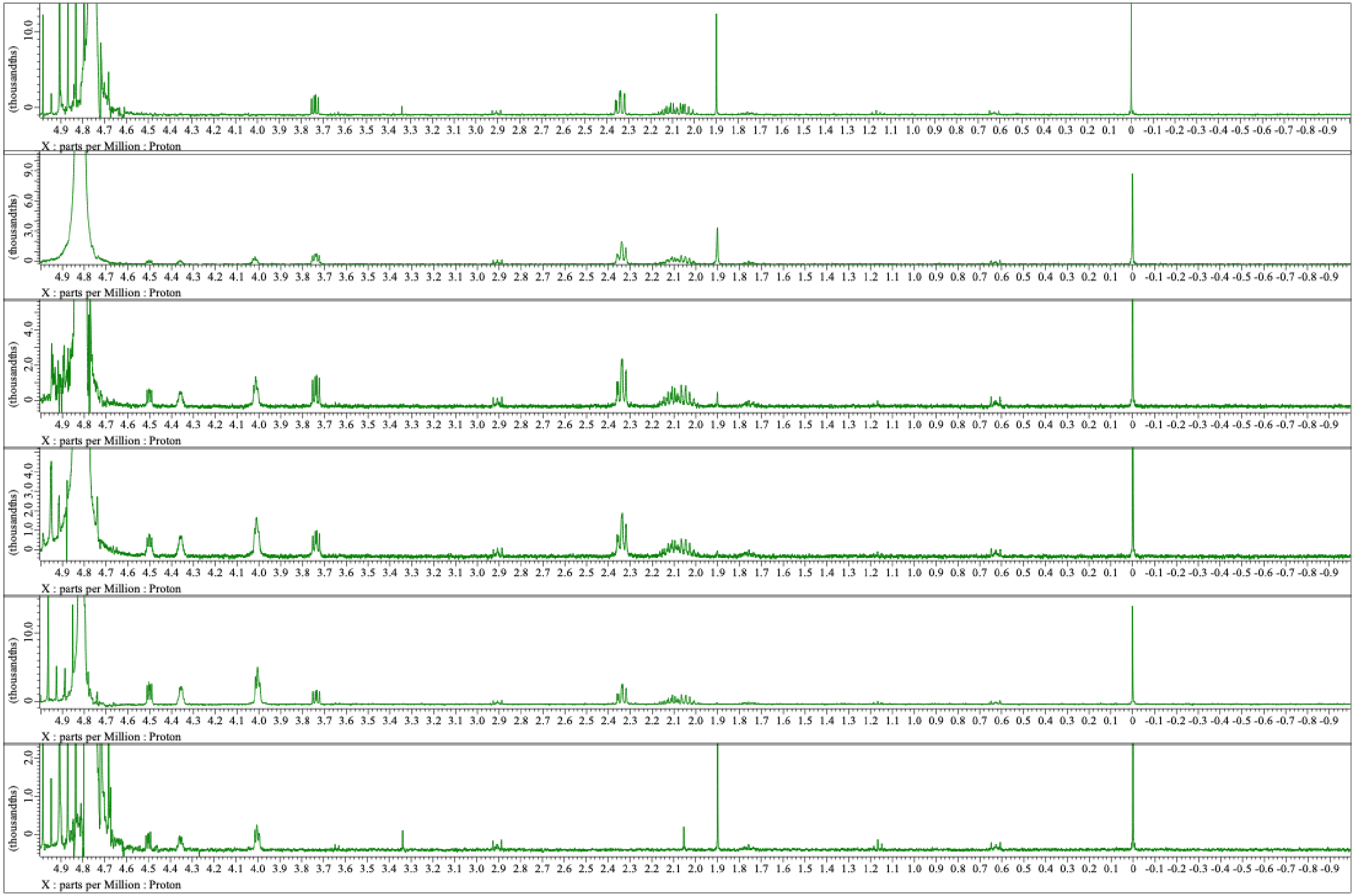

Figure S7. Expanded spectrum [δ < 5 ppm] (MSG：IMP)
